# Loss of TROP2 and epithelial cell adhesion molecule expression is linked to grade progression in pTa but unrelated to disease outcome in pT2-4 urothelial bladder carcinomas

**DOI:** 10.3389/fonc.2023.1342367

**Published:** 2024-01-12

**Authors:** Jan H. Müller, Henning Plage, Sefer Elezkurtaj, Tim Mandelkow, Zhihao Huang, Magalie C. J. Lurati, Jonas B. Raedler, Nicolaus F. Debatin, Eik Vettorazzi, Henrik Samtleben, Sebastian Hofbauer, Kira Furlano, Jörg Neymeyer, Irena Goranova, Bernhard Ralla, Sarah Weinberger, David Horst, Florian Roßner, Simon Schallenberg, Andreas H. Marx, Margit Fisch, Michael Rink, Marcin Slojewski, Krystian Kaczmarek, Thorsten Ecke, Steffen Hallmann, Stefan Koch, Nico Adamini, Maximilian Lennartz, Sarah Minner, Ronald Simon, Guido Sauter, Henrik Zecha, Thorsten Schlomm, Elena Bady

**Affiliations:** ^1^ Institute of Pathology, University Medical Center Hamburg-Eppendorf, Hamburg, Germany; ^2^ Department of Urology, Charité Berlin, Berlin, Germany; ^3^ College of Arts and Sciences, Boston University, Boston, MA, United States; ^4^ Department of Medical Biometry and Epidemiology, University Medical Center Hamburg-Eppendorf, Hamburg, Germany; ^5^ Department of Urology, Academic Hospital Fuerth, Fuerth, Germany; ^6^ Insitute of Pathology, Charité Berlin, Berlin, Germany; ^7^ Department of Pathology, Academic Hospital Fuerth, Fuerth, Germany; ^8^ Department of Urology, University Medical Center Hamburg-Eppendorf, Hamburg, Germany; ^9^ Department of Urology, University Hospital Stettin, Stettin, Poland; ^10^ Department of Urology, Helios Hospital Bad Saarow, Bad Saarow, Germany; ^11^ Department of Pathology, Helios Hospital Bad Saarow, Bad Saarow, Germany; ^12^ Department of Urology, Albertinen Hospital, Hamburg, Germany

**Keywords:** TROP2, EpCAM, muscle invasive urothelial cancer, multiplex fluorescence immunohistochemistry, bladder cancer

## Abstract

**Introduction:**

Trophoblast cell surface antigen 2 (TROP2; EpCAM2) is a transmembrane glycoprotein which is closely related to EpCAM (EpCAM; EpCAM1). Both proteins share partial overlapping functions in epithelial development and EpCAM expression but have not been comparatively analyzed together in bladder carcinomas. TROP2 constitutes the target for the antibody-drug conjugate Sacituzumab govitecan (SG; TrodelvyTM) which has been approved for treatment of metastatic urothelial carcinoma by the United States Food and Drug administration (FDA) irrespective of its TROP2 expression status.

**Methods:**

To evaluate the potential clinical significance of subtle differences in TROP2 and EpCAM expression in urothelial bladder cancer, both proteins were analyzed by multiplex fluorescence immunohistochemistry in combination with a deep-learning based algorithm for automated cell detection on more than 2,700 urothelial bladder carcinomas in a tissue microarray (TMA) format.

**Results:**

The staining pattern of TROP2 and EpCAM were highly similar. For both proteins, the staining intensity gradually decreased from pTa G2 low grade (TROP2: 68.8±36.1; EpCAM: 21.5±11.7) to pTa G2 high grade (64.6±38.0; 19.3±12.2) and pTa G3 (52.1±38.7; 16.0±13.0, p<0.001 each). In pT2-4 carcinomas, the average TROP2 and EpCAM staining intensity was intermediate (61.8±40.9; 18.3±12.3). For both proteins, this was significantly lower than in pTa G2 low grade (p<0.001 each) but also higher than in pTa G3 tumors (p=0.022 for TROP2, p=0.071 for EpCAM). Within pT2-4 carcinomas, the TROP2 and EpCAM staining level was unrelated to pT, grade, UICC-category, and overall or tumor-specific patient survival. The ratio TROP2/EpCAM was unrelated to malignant phenotype and patient prognosis.

**Conclusion:**

Our data show that TROP2 and EpCAM expression is common and highly interrelated in urothelial neoplasms. Despite of a progressive loss of TROP2/EpCAM during tumor cell dedifferentiation in pTa tumors, the lack of associations with clinicopathological parameters in pT2-4 cancer argues against a major cancer driving role of both proteins for the progression of urothelial neoplasms.

## Introduction

Urinary bladder cancer is the tenth most frequent cancer worldwide and the sixth leading cause of death by cancer in men (1). Low-grade non-invasive (pTa) or minimally-invasive (pT1) tumors are present in 80% of bladder cancer patients but can be removed by transurethral resection (TUR-B) and show a good prognosis (2). In patients with muscle-invasive bladder cancer, treatment usually consists of neoadjuvant chemotherapy plus radiotherapy or radical cystectomy, but outcomes remain variable and almost 50% of the patients develop early metastasis and eventually die from their disease (3).

The TROP2 (Trophoblast cell surface antigen 2)-directed antibody-drug conjugate Sacituzumab govitecan (SG; Trodelvy™) is a new therapeutic option for bladder cancer patients with metastatic disease (4). TROP2, also named EpCAM2 is a transmembrane glycoprotein with a role for intracellular calcium signaling, proliferation, transformation, cell self-renewal and is expressed in many normal tissues (5). TROP2 is overexpressed in many cancers, can promote tumor growth and is of prognostic relevance (6–8). Sacituzumab govitecan (SG) has been approved for treatment of metastatic triple negative breast cancer (9) and metastatic urothelial carcinomas (10). Patients obtain SG treatment irrespective of the expression level of TROP2. Patients with moderate to strong TROP2 overexpression showed a particularly high response to therapy whereas some studies also revealed a response in tumors with low TROP2 expression (11, 12).

The homologues molecule to TROP2 is the epithelial cell adhesion molecule (EpCAM; EpCAM1) which plays a pivotal role in embryonic stem cell proliferation, differentiation, migration as well as epithelial mesenchymal transition (EMT) and may contribute to cell adhesion in normal and neoplastic epithelial cells (13, 14). Data from several studies are proposing at least partial overlapping functions in regards to epithelial development (15) (16). Although TROP2 and EpCAM are both highly expressed in most urothelial neoplasms, the level of expression might be relevant. However, the findings of studies comparing EpCAM or TROP2 expression with clinico-pathological features were discrepant. Several studies described associations between high EpCAM/TROP2 expression and poor bladder cancer prognosis (17–20) while others found a relationship with favorable tumor features in adenocarcinoma of non-small cell lung carcinoma (21) and upper tract urothelial carcinoma (22). Difficulties in the quantification of highly expressed proteins in brightfield immunohistochemistry (IHC) may constitute a reason for some of these discrepant data. Moreover, studies evaluating the structurally and functionally related TROP2 and EpCAM proteins in combination are so far lacking.

To study the biological significance and potential clinical role of different levels of TROP2 and EpCAM, more than 2,700 tumor samples were analyzed for TROP2 and EpCAM expression by multiplex fluorescence immunohistochemistry in combination with a deep-learning based algorithm for automated cell detection in a tissue microarray (TMA) format. This approach was also based on the assumption that fluorescence immunohistochemistry (IHC) may enable a subtler quantification of protein expression than brightfield IHC.

## Materials and methods

### Tissue microarrays (TMA)

Our set of TMAs contained one sample each from 2,768 urothelial tumors of the bladder archived at the Institute of Pathology, University Hospital Hamburg, Institute of Pathology, Charité Berlin, Department of Pathology, Academic Hospital Fuerth, or Department of Pathology, Helios Hospital Bad Saarow, and/or treated at Department of Urology, University Hospital Hamburg, Department of Urology, Charité Berlin, Department of Urology, Helios Hospital Bad Saarow, Department of Urology, Albertinen Hospital Hamburg (all in Germany), and Department of Urology and Urological Oncology, Pomeranian Medical University, Szczecin, Poland between 2003 and 2021. Patients at each center were treated according to the guidelines at the time. In brief, patients with pTa/pT1 disease underwent a transurethral resection of the bladder tumor with or without postoperative instillation therapy, while 459 of 2,768 patients with pT2-pT4 disease were treated by radical cystectomy between 2003 and 2016. Available histopathological data including grade, tumor stage (pT), lymph node status (pN), and status of venous (V) and lymphatic (L) invasion are shown in **Table 1**. Clinical follow up data for patient’s overall survival (OS) was available from 592 patients and from 235 patients for cancer specific survival (CSS) within pT2-4 carcinomas treated by cystectomy (median follow-up time: 15 months; range: 1-176 months). The tissues were fixed in 4% buffered formalin and then embedded in paraffin. The TMA manufacturing process has previously been described in detail (23, 24). In brief, one tissue spot (diameter: 0.6 mm) was transmitted from a cancer containing donor block into an empty recipient paraffin block. The use of archived remnants of diagnostic tissues for TMA manufacturing, their analysis for research purposes, and patient data were according to local laws (HmbKHG, §12) and analysis had been approved by the local ethics committee (Ethics commission Hamburg, WF-049/09). All work has been carried out in compliance with the Helsinki Declaration.

**Table 1:** Patient characteristics.

Patients characteristics	No. of patients (%)
	Total study cohort on TMA (n=2768)
Follow-up - no. (%)	635 (22.9%)
Median - months (95% confidence interval)	15 (13 - 17)
Sex	
Male	1819 (65.7%)
Female	547 (19.8%)
Missing data	402 (14.5%)
pT stage - no. (%)	
pTa G2 low	460 (16.6%)
pTa G2 high	226 (8.2%)
pTa G3	198 (7.2%)
pT1	49 (1.8%)
pT2	462 (16.7%)
pT3	615 (22.2%)
pT4	298 (10.8%)
Missing data	460 (16.6%)
pN stage - no. (%)	
pN-	734 (26.5%)
pN+	449 (16.2%)
Missing data	1585 (57.3%)
R status - no. (%)	
R-	595 (21.5%)
R+	143 (5.2%)
Missing data	2030 (73.3%)
L status - no. (%)	
L-	275 (9.9%)
L+	281 (10.2%)
Missing data	2212 (79.9%)
V status - no. (%)	
V-	450 (16.3%)
V+	155 (5.6%)
Missing data	2163 (78.1%)
Grade - no. (%)	
2	820 (29.6%)
3	1858 (67.1%)
Missing data	90 (3.3%)

### Immunohistochemistry (IHC)

For multiplex fluorescence immunostaining (mfIHC) freshly cut 4µm consecutive TMA sections and the OPAL dye kit (Cat. # NEL811001KT, AKOYA Biosciences, Menlo Park, California, United States) were used. The experimental procedure was performed as previously described (25). Slides were deparaffinized and treated with heat-induced antigen retrieval pH 7.8 buffer in an autoclave for 5 min at 100-121°C. Primary antibodies specific for Cytokeratin Pan (MSVA-000R), EpCAM (MSVA-326R) and TACSTD2/TROP2 (MSVA-733R, MS Validated Antibodies GmbH) were applied at 37°C for 60 min on one day. Secondary anti rabbit antibody was applied and bound antibody was visualized using the EnVision Kit (Dako; Agilent Technologies, Inc.) according to the manufacturer’s directions. For details of the used reagents see **Table 2**.

**Table 2:** List of the used antibody clones, antigen retrieval (AR), dilutions, staining positions and opal dyes for multiplex fluorescence immunohistochemistry.

Antibody target	Identifier	AR (pH value)	Dilution	Staining position	Opal dye
panCK	MSVA, Clone: MSVA-000R Cat#: 2105-000R-05	7.8	1:1800	1	520
TROP2	MSVA, Clone: MSVA-733R Cat#: 3648-733R-05	7.8	1:1200	2	570
EpCAM	MSVA, Clone: MSVA-326R Cat#: 2315-326R-05	7.8	1:2400	3	690

(MSVA, MS Validated Antibodies GmbH; AR, antigen retrieval).

### Analysis of multiplex fluorescence immunohistochemistry (mfIHC) images

A Leica Aperio VERSA 8 automated epifluorescence microscope was used for digitizing mfIHC stained slides with intensity measurements for each of the used OPAL fluorophores (range 0-255). Image analysis was performed by using a pretrained deep learning-based (U-Net) approach for cell detection, cell segmentation and intensity measurement of the used OPAL fluorophores (range 0-255), processing the intensity values using python version 3.8 (26), R version 3.6.1 (The R foundation) (27) and the Visiopharm software package (Hoersholm, Denmark). The intensity of each fluorochrome was recorded as the raw intensity for each individual cell. The image analysis workflow has been described in detail before (25). A cutoff for panCK positive cells were set to ≥12 to only consider panCK positive cells as cancer cells for this study. For calculating the EpCAM/TROP2 ratio a cutoff was set to ≥1 for both marker before ratio calculation, to avoid excessive ratios.

### Statistics

All statistic calculations were performed with R version 3.6.1 (The R foundation) and JMP Pro 16 software package (SAS Institute Inc., NC, USA (JMP^®^) (28). Contingency tables and the ANOVA were performed to search for associations between EpCAM and TROP2 immunostaining and tumor phenotype of selected tumor types and subtypes. Kaplan-Meier survival curves were calculated according to the overall survival of each patient. The Log-Rank test was used to detect significant differences between groups. A p-value of ≤0.05 was considered statistically significant.

## Results

### Technical issues

**Figure 1:**
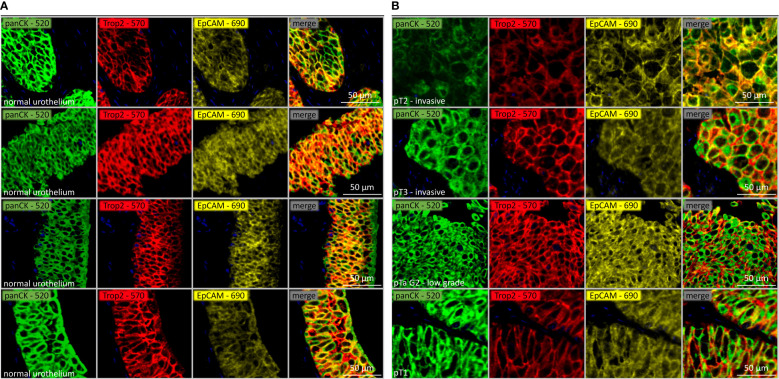
**(A)** TROP2 (red), EpCAM (yellow) and combined staining intensity (co-expression) on normal urothelial tissue. Epithelial cells are displayed with panCK (green). **(B)** TROP2 (red), EpCAM (yellow) and combined staining intensity (co-expression) in pTa and muscle invasive urothelial carcinomas. Epithelial cells are displayed with panCK (green).

Of our 2,768 urothelial carcinomas, 2,580 (93.2%) were interpretable for both TROP2 and EpCAM. Representative images of TROP2, EpCAM and combined staining is shown in **Figure 1A** for normal urothelial tissue and in **Figure 1B** for urothelial carcinomas. Non-interpretable tumors were caused by a lack of unequivocal (panCK positive) tumor cells on the TMA spots or the complete absence of the entire tissue at the respective TMA spot.

### TROP2 in urothelial carcinomas

**Figure 2:**
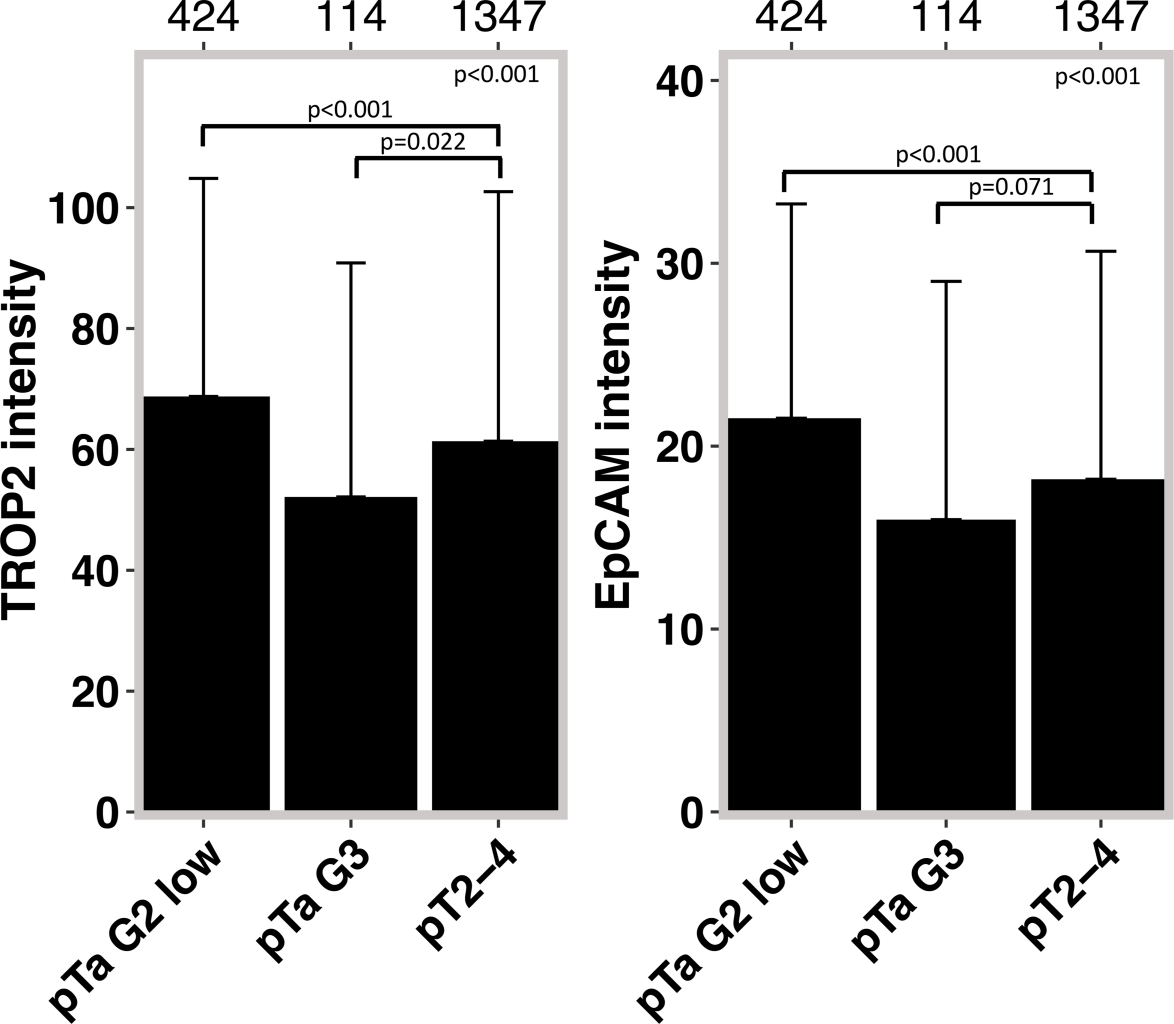
TROP2 and EpCAM intensity in pTa G2 low, pTa G3 and muscle invasive (pT2-4) bladder cancer.

**Figure 3:**
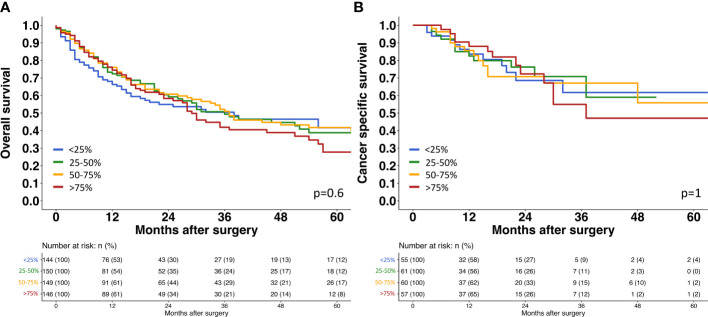
Survival assessment of TROP2 expression in muscle invasive urothelial bladder cancer. **(A)** Survival assessment of Trop2 expression according to overall survival. **(B)** Survival assessment of Trop2 expression according to cancer specific survival.

A strong TROP2 staining was always seen in normal urothelial cells (46.4 ± 44.5). The relationship between TROP2 staining intensity and tumor phenotype is shown in **Table 3**. Within pTa tumors, the highest TROP2 staining intensity was seen in pTa G2 low grade tumors. The staining intensity gradually decreased from pTa G2 low grade (68.8 ± 36.1) to pTa G2 high grade (64.6 ± 38.0) and pTa G3 (52.1 ± 38.7; p<0.001). In 1,375 pT2-4 carcinomas, the average TROP2 staining intensity was intermediate. It was significantly lower than in pTa G2 low grade (p<0.001) but also significantly higher than in pTa G3 tumors (p=0.022) (**Figure 2**). Within pT2-4 carcinomas, the TROP2 staining level was unrelated to pT (p=0.3), grade (p=0.9), pN (p=0.12), R-status (p=0.2), V-status (p=0.069) and UICC-category (p=0.5) but significantly linked to L1 status (p<0.001). TROP2 staining levels were unrelated to overall and tumor specific survival (**Figure 3**).

**Table 3:** TROP2 and EpCAM immunostaining of normal urothel, pTa and muscle invasive cancers.

		EpCAM			TROP2			TROP2/EpCAM (cutoff =1)		
		n	Mean ± SD	p	n	Mean ± SD	p	n	Mean ± SD	p
only pT2-4	normal Urothel	51	14.2 ± 14.5	0.073*	51	46.4 ± 44.5	0.036*	40	3.3 ± 1.1	0.095*
				<0.001**			<0.001**			0.6**
	pTa G2 low	424	21.5 ± 11.7	<0.001	424	68.8 ± 36.1	<0.001	408	3.4 ± 1.2	<0.001
	pTa G2 high	196	19.3 ± 12.2		196	64.6 ± 38.0		185	4.0 ± 2.4	
	pTa G3	114	16.0 ± 13.0		114	52.1 ± 38.7		94	3.7 ± 2.1	
	pT2	451	18.7 ± 12.6	0.3	451	62.4 ± 41.8	0.3	413	3.7 ± 2.0	0.3
	pT3	602	17.6 ± 12.7		602	59.4 ± 41.9		541	3.8 ± 1.9	
	pT4	294	18.5 ± 11.7		294	63.6 ± 39.1		277	3.9 ± 1.9	
	G2	107	17.6 ± 12.6	0.6	107	60.8 ± 42.3	0.9	97	4.0 ± 1.9	0.2
	G3	1213	18.3 ± 12.5		1213	61.5 ± 41.3		1109	3.8 ± 1.9	
	pN0	669	17.3 ± 12.5	0.045	669	58.8 ± 41.3	0.12	599	3.9 ± 2.1	0.3
	pN+	441	18.8 ± 12.2		441	62.8 ± 40.0		409	3.7 ± 1.8	
	R0	560	17.0 ± 13.1	0.4	560	59.1 ± 43.6	0.2	491	3.9 ± 2.3	0.7
	R1	139	17.9 ± 11.9		139	64.1 ± 39.8		130	4.0 ± 1.8	
	V0	429	16.8 ± 12.5	0.093	429	59.4 ± 42.2	0.069	379	3.9 ± 2.2	0.7
	V1	154	18.8 ± 12.7		154	66.7 ± 43.1		142	4.0 ± 2.1	
	L0	263	14.5 ± 12.5	<0.001	263	51.1 ± 41.2	<0.001	217	4.0 ± 2.5	0.6
	L1	275	19.2 ± 12.5		275	67.8 ± 42.4		261	3.9 ± 2.0	
	UICC I-II	15	17.6 ± 15.1	0.5	15	55.7 ± 41.6	0.5	11	3.7 ± 1.7	0.7
	UICC III	61	16.7 ± 11.1		61	66.9 ± 46.4		58	4.3 ± 2.5	
	UICC IV	49	14.7± 8.8		49	59.8 ± 38.8		48	4.2 ± 2.1	

*vs. all tumors, **vs. pTa G2 low. (SD, Standard deviation).

### EpCAM in urothelial carcinomas

**Figure 4:**
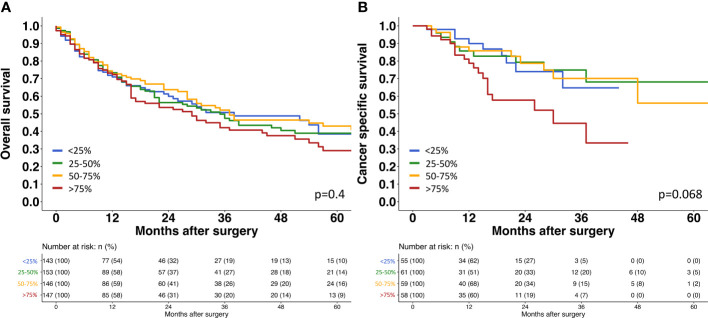
Survival assessment of EpCAM expression in muscle invasive urothelial bladder cancer. **(A) **Survival assessment of EpCAM expression according to overall survival. **(B)** Survival assessment of EpCAM expression according to cancer specific survival.

The relationship of EpCAM expression with tumor phenotype was highly similar as seen for TROP2 (**Table 3**). EpCAM staining was always intense in normal urothelial cells (14.2 ± 14.5) and - among tumors - it was highest in pTa G2 low grade neoplasms. EpCAM staining decreased from pTa G2 low grade (21.5 ± 11.7) to pTa G2 high grade (19.3 ± 12.2) and pTa G3 (16 ± 13; p<0.001). In 1,375 pT2-4 carcinomas, the average EpCAM staining intensity was again intermediate (18.3 ± 12.3) and ranged between the values seen in pTa G2 low grade (p<0.001 for pTa G2 low grade vs. pT2-4) and pTa G3 tumors (p=0.071 for pTa G2 high grade vs. pT2-4). In pT2-4 carcinomas, EpCAM staining was unrelated to grade (p=0.6), pT (p= 0.3), R-status (p=0.4) but significantly linked to pN (p=0.045) and L1 status (p<0.001). Associations were not seen between EpCAM staining levels and overall or tumor specific survival (**Figure 4**).

### TROP2/EpCAM ratio in urothelial carcinomas

**Figure 5:**
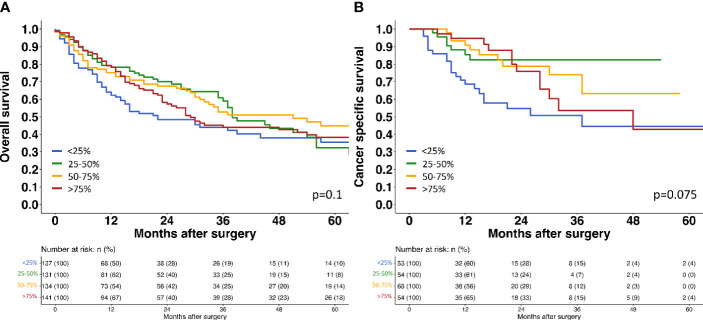
Survival assessment of TROP2/EpCAM ratio in muscle invasive urothelial bladder cancer. **(A)** Survival assessment of Trop2/EpCAM ratio according to overall survival. **(B)** Survival assessment of Trop2/EpCAM ratio according to cancer specific survival.

The TROP2/EpCAM ratio was largely unrelated to tumor phenotype (**Table 2**). Within pTa tumors, the TROP2/EpCAM ratio was higher in pTa G2 high grade tumors (4.0 ± 2.4) than in pTa G2 low grade (3.4 ± 1.2) or in pTa G3 tumors (3.7 ± 2.1; p<0,001). Within muscle-invasive cancers, the TROP2/EpCAM ratio marginally changed from pT2 (3.7 ± 2) to pT3 (3.8 ± 1.9) and pT4 (3.9 ± 1.9) cancers, but these differences did not reach statistical significance (p=0.3). The TROP2/EpCAM ratio was also unrelated to overall and tumor specific survival in pT2-4 carcinomas (**Figure 5**).

## Discussion

Our analysis of more than 2700 urothelial carcinomas revealed a frequent and mostly high-level expression of both TROP2 and EpCAM in non-invasive and invasive urothelial carcinomas. This is in line with the majority of previous studies. Three earlier IHC studies had found TROP2 positivity in 93-100% of muscle-invasive urothelial carcinomas (average 96% TROP2 positive) (22, 29, 30). EpCAM expression was found in 44-93% muscle-invasive urothelial carcinomas (average 61% EpCAM positive) (17, 31–33). Considering the high frequency of TROP2 and/or EpCAM positive urothelial carcinomas and the low dynamic range of brightfield immunohistochemistry for protein quantification, immunofluorescence was used for the quantification of our IHC results in this study to search for a clinical relevance of subtle expression differences. In contrast to brightfield IHC, immunofluorescence enables a more sensitive quantification of different levels of protein expression (34, 35). In our cohort of more than 800 pTa tumors, the analysis revealed a significant decrease from pTa G2 low-grade to pTa G2 high-grade, and pTa G3 tumors for both proteins. It is of note that similar observations were recorded by brightfield immunohistochemistry in analyses of partially overlapping sets of pTa tumors in separate studies determining the expression of TROP2 and EpCAM in >10,000 tumors from up to 150 different tumor entities (30, 33). In these studies, the same reagents were used which have previously been validated according to the recommendations of the international working group for antibody validation (IWGAV) (36) by comparison with independent antibodies and with RNA expression data in 76 different normal tissue categories.

The continuous decrease of TROP2 and EpCAM expression with grade in non-invasive urothelial bladder carcinomas in combination with the somewhat higher expression levels – to the degree of pTa G2 – in pT2-4 carcinomas could be explained by the unique evolution of pTa bladder cancers *in vivo*. Non-invasive urothelial neoplasms tend to diffusely disseminate within the bladder and the upper urinary tract (37). Resection of papillary tumors is thus often incomplete and clonally related tumor remnants frequently remain in the bladder as invisible flat lesions or minor papillary tumors which serve as a source for a multitude of subsequent recurrences (38). Comparable to the situation in tumor cell lines *in vitro*, non-invasive urothelial neoplasms can thus continuously accumulate genomic alterations over a long period of time (39). In many patients, pTa tumor evolution is only terminated if the neoplastic cells acquire the capability of invasive tumor growth which may eventually terminate genomic tumor progression by either the surgical removal of the urinary bladder or the cancer related death of the patient. As in other tumor entities, the accumulation of genomic alterations results in an increasing degree of cellular atypia and a reduced expression of a continuously growing number of physiologically expressed genes – such as TROP2 and EpCAM - in high grade tumors (40, 41).

Considering the close structural and potentially also functional relationship between TROP2 (EpCAM2) and EpCAM the clinical impact of the TROP2/EpCAM ratio was analyzed in this study. The complete lack of associations between the TROP2/EpCAM ratio and histopathological or clinical data – except in the case of the L-status - strongly argues against a potential biologic role of a disbalance of these two structurally related proteins. A parallel expression of both proteins is consistent with reports by Szabo et al. showing at least partially overlapping expression patterns and functions of EpCAM and TROP2 as regulators of epithelial development and a shared role in claudin stabilization (15). Further research pointing to shared functions, showing that only a combined knockout of both EpCAM and TROP2 was leading to a dramatic decrease of claudin levels in cultured human keratinocytes (42–44). Furthermore, it has been shown that TROP2 was able to compensate for the loss of EpCAM in stabilizing claudin-7 expression and cell membrane localization in tissues that co-express both proteins (15). Those functional similarities are underlined by the previously reported 49% sequence identity and 67% sequence similarity between these two proteins (5, 45).

The complete absence of associations between TROP2/EpCAM expression – even if measured with a highly quantitative method - and histopathological parameters of cancer aggressiveness or clinical outcome in muscle-invasive urothelial carcinoma is also consistent with RNA data from The Cancer Genome Atlas (TCGA) (https://www.cancer.gov/tcga). In combination these findings strongly argue against a major cancer driving role of TROP2/EpCAM activation (or inactivation) in muscle-invasive urothelial carcinoma. This notion is consistent with controversial data on the prognostic role of TROP2 expression in other tumor entities. Although, high expression of TROP2 has been linked to poor patient prognosis in pancreatic cancer (46), prostate cancer (47), oral squamous cell carcinomas (48), gastric cancer (49), colon cancer (50, 51), cervical cancer (52), gallbladder cancer (53) and ovarian cancer (54), while there were other studies describing associations between low TROP2 expression and poor patient prognosis in lung cancer (21, 54). Absence of a strong role of TROP2 for cancer progression is to some extent counterintuitive because some successful target proteins of cancer drugs such as HER2 are established drivers of cancer aggressiveness (55, 56). However, other well-established drug target proteins such as CD20 (57), CD30 (58), CD52 (59), SLAMF7 (60), CD38 (61), GD2 (62) also lack significant evidence for a driving role in cancer progression.

In summary, our data show that TROP2 and EpCAM expression is common and highly interrelated in urothelial neoplasms. Declining levels of expression for both proteins with increasing tumor grade is consistent with a progressive loss of TROP2/EpCAM during tumor cell dedifferentiation. However, the lack of associations with clinic-pathological parameters in muscle-invasive cancer argues against a major cancer driving role of both proteins in urothelial neoplasms.

## Data availability statement

The raw data supporting the conclusions of this article will be made available by the authors, without undue reservation.

## Ethics statement

The studies involving humans were approved by Ethics commission Hamburg, WF-049/09. The studies were conducted in accordance with the local legislation and institutional requirements. The human samples used in this study were acquired from a by- product of routine care or industry. Written informed consent for participation was not required from the participants or the participants’ legal guardians/next of kin in accordance with the national legislation and institutional requirements.

## Author contributions

JM: Formal analysis, Validation, Writing – original draft. HP: Writing – review & editing. SE: Writing – review & editing. TM: Writing – review & editing. ZH: Writing – review & editing. MCJL: Writing – review & editing. JR: Methodology, Software, Writing – review & editing. ND: Writing – review & editing. EV: Data curation, Writing – review & editing. HS: Writing – review & editing. SHo: Writing – review & editing. KF: Writing – review & editing. JN: Writing – review & editing. IG: Writing – review & editing. BR: Writing – review & editing. SW: Writing – review & editing. DH: Writing – review & editing. FR: Writing – review & editing. SS: Writing – review & editing. AM: Writing – review & editing. MF: Writing – review & editing. MR: Writing – review & editing. MS: Writing – review & editing. KK: Writing – review & editing. TE: Writing – review & editing. SHa: Writing – review & editing. SK: Writing – review & editing. NA: Writing – review & editing. ML: Writing – review & editing. SM: Writing – review & editing. RS: Writing – review & editing. GS: Writing – review & editing, Conceptualization, Project administration, Supervision. HZ: Writing – review & editing. TS: Writing – review & editing. EB: Data curation, Formal analysis, Project administration, Supervision, Validation, Writing – original draft, Writing – review & editing.
